# Liddle-Mutation of the β-Subunit, but not the γ-Subunit, Attenuates Protein Kinase C-Mediated Inhibition of Human Epithelial Sodium Channels (hENaC)

**DOI:** 10.1007/s00232-015-9866-x

**Published:** 2016-01-12

**Authors:** Gerard G. Robins, Geoffrey I. Sandle

**Affiliations:** Leeds Institute of Biomedical and Clinical Sciences, St James’s University Hospital, Level 7, Clinical Sciences Building, Beckett Street, Leeds, LS9 7TF UK

**Keywords:** ENaC, Liddle mutations, Oocytes, Protein kinase C, Sodium channels

## Abstract

Mammalian distal nephron and distal colon, prime sites for Na^+^ homeostasis, contain amiloride-sensitive epithelial sodium channels (ENaC). Protein kinase C (PKC) inhibits ENaC by phosphorylating serine and threonine residues within COOH termini of the β- and/or γ-subunits. Although some of these phosphorylation sites are close to the PY motifs, it is unclear whether they remain susceptible to PKC in Liddle-mutated ENaC β- and/or γ-subunits, where PY motifs are truncated, resulting in increased apical ENaC expression. We therefore studied the effects of PKC in wild-type and Liddle-mutated human epithelial Na^+^ channels (hENaC) expressed in *Xenopus* oocytes, using the dual-electrode voltage clamp technique. PKC activation using 500 nmol/l phorbol 12-myristate 13-acetate (PMA) decreased amiloride-sensitive Na^+^ currents by 80 % in oocytes expressing wild-type hENaC, an effect largely prevented by co-exposure to 50 µmol/l calphostin C (a specific inhibitor of PKC), whereas 500 nmol/l phorbol didecanoate (PDD), an inactive phorbol ester which does not stimulate PKC, had no effect. In oocytes expressing hENaC containing the Liddle-mutated β-subunit, PMA elicited a 54 % decrease in amiloride-sensitive Na^+^ currents, significantly (*P* < 0.0025) less than that in oocytes expressing wild-type hENaC. By contrast, in oocytes expressing hENaC containing the Liddle-mutated γ-subunit, PMA elicited a 68 % decrease in amiloride-sensitive Na^+^ current, similar (*P* = 0.10) to that in oocytes expressing wild-type hENaC. We conclude that hENaC incorporating the Liddle-mutated β-subunit lacks one or more PKC phosphorylation sites, thereby significantly reducing the inhibitory effect of PKC on Na^+^ channel activity, whereas hENaC incorporating Liddle-mutated γ-subunits remains as susceptible to PKC as wild-type hENaC.

## Introduction

Apical membrane Na^+^ channels (ENaC) are expressed in many Na^+^ absorptive epithelia, including the distal nephron, distal colon, lung, urinary bladder, salivary gland ducts, and sweat glands. The distal nephron and distal colon in particular function as “Na^+^ scavengers,” and have important roles in maintaining Na^+^ (and thus water) homeostasis. In these epithelia, the rapid up-regulation of ENaC is one of the mechanisms invoked to compensate for the fluid losses that occur secondary to hemorrhage, infective and other types of secretory diarrhea, high-output ileostomies, and during periods of sustained exercise and fluid restriction. Such rapid responses are dependent on a number of extrinsic (e.g., aldosterone and arginine vasopressin) and intrinsic (e.g., ENaC trafficking, deubiquitination, and protein kinase A-mediated phosphorylation) factors, which increase ENaC synthesis, expression, and/or function (Bhalla and Hallows 2008). However, basal ENaC activity is also tightly regulated to prevent volume expansion and hypertension, and protein kinase C (PKC)-mediated inhibition of ENaC expression and activity via the mitogen-activated protein kinase/ERK pathway is one potential mechanism (Zeissig et al. 2008). Studies in a variety of epithelia and planar lipid bilayers have shown that the activation of PKC (a Ca^2+^- and phospholipid-dependent protein kinase) reduced ENaC activity by decreasing both open channel probability (Ling and Eaton 1989; Ling et al. 1992; Oh et al. 1993; Awayda et al. 1996) and channel density (Els et al. 1998). In renal A6 cells, the PKC-mediated decrease in ENaC activity appeared to reflect effects on ENaC β- and γ-subunits, but not the α-subunit (Stockand et al. 2000). Phorbol 12-myristate 13-acetate (PMA), a phorbol ester that activates PKC, also stimulated phosphorylation of rat ENaC β- and γ-subunits (Shimkets et al. 1998). Human, rat, and *Xenopus* ENaC possess five conserved phosphorylation sites, one on each COOH-terminus near the PY motif of the β- and γ-subunits, and one on each NH_2_-terminus of both subunits, although the COOH-terminus of the β-subunit appears to be a poor substrate for PKC-mediated phosphorylation (McDonald et al. 1995; Barbry and Hofman 1997). In addition, the COOH-terminus of human ENaC γ-subunit is predicted to have a strong PKC phosphorylation site (McDonald et al. 1995).

Some people of African descent with salt-sensitive hypertension possess a mutation involving the replacement of threonine by methionine at position 594 (β-T594M) in the PKC consensus site of the ENaC β-subunit, which appears to be unrelated to the Liddle mutation of the β-subunit (Cui et al. 1997). Lymphocytes from these patients exhibited greater whole-cell Na^+^ currents in response to the membrane-permeant cAMP analog 8-(4-chlorophenylthio) adenosine 3′,5′-cyclic monophosphate (8-cpt-cAMP) than lymphocytes from normotensive individuals expressing wild-type ENaC. Furthermore, PMA abolished 8-cpt-cAMP-stimulated Na^+^ channel activity in lymphocytes expressing wild-type ENaC, whereas PMA had no effect in lymphocytes with homozygotic mutations, and heterozygotes exhibited an intermediate effect (Cui et al. 1997). The putative PKC phosphorylation site at position 594 is outside the PY motif, and while lymphocytes expressing the Liddle-mutated PY motif had larger basal Na^+^ currents than controls, 8-cpt-cAMP had no additional stimulatory effect (Bubien et al. 1996). Since PKC phosphorylation sites exist near the PY motifs of the γ-subunit as well as the β-subunit (Barbry and Hofman 1997), it is conceivable that Liddle-mutated β- and γ-subunits have defective PKC consensus sites, resulting in loss of a mechanism for down-regulating Na^+^ channels. Thus, the aim of the present study was to evaluate the effect of PKC on amiloride-sensitive Na^+^ currents in *Xenopus* oocytes expressing wild-type human ENaC (hENaC), Liddle-mutated hENaC β-subunit alone, Liddle-mutated hENaC γ-subunit alone, or Liddle-mutated hENaC β-subunit and Liddle-mutated hENaC γ-subunit in combination.

## Methods

### Preparation of cDNA Constructs and Microinjection into *Xenopus* Oocytes

hENaC subunit cDNAs were incorporated into pMT3 vector (a gift of Dr. P. Snyder, University of Iowa, USA). The vector contained one of the three wild-type subunits, the β-subunit with a Liddle-type truncation (β566X), or the γ-subunit with a Liddle-type truncation (γ576X). Clones were amplified by transforming competent *Escherichia coli* grown on LB-ampicillin agar plates, the pMT3 vector being ampicillin resistant. Plasmids were prepared using a proprietary kit (QIAGEN). Female *Xenopus laevis* (European Xenopus Resource Centre, University of Portsmouth, Portsmouth, UK) were killed by a schedule 1 method approved by the UK Home Office. Ovaries were removed, washed in modified Barth’s saline (MBS), and divided into clumps of 10–30 oocytes, which were separated using Ca^2+^-free Ringer’s solution containing 1 mg/ml collagenase, as described previously (Canessa et al. 1993). Oocytes at Dumont stages V and VI were transferred to 96-well plates containing MBS, centrifuged (2100 rpm, 15 min), and the nuclei microinjected with either 20 nl of sterile distilled water, or 20 nl of sterile distilled water containing (3.5 ng of each subunit cDNA) wild-type hENaC, hENaC with the Liddle-mutated β-subunit, or hENaC with the Liddle-mutated γ-subunit. Injected oocytes were transferred to 24-well plates containing MBS (96 mmol/l Na^+^) and incubated at 19 °C for 24–48 h.

### Dual-Electrode Voltage Clamp Recording

Oocytes were superfused (1 ml/min) with a solution containing (in mmol/l): Na^+^ gluconate 100, Ca^2+^ 0.38, Mg^2+^ 0.47, Cl^−^ 11.7, and HEPES 4.6 (pH 7.4), with Ba^2+^ 5.0 and tetraethylammonium 10 to block endogenous K^+^ channels. Oocytes were impaled with the voltage and current electrodes (tip resistances <1 MΩ) fabricated from glass microcapillary tubing and back-filled with 3 mol/l KCl. Experiments were done at room temperature (20–22 °C). When membrane voltage was stable, command voltages (−140 to +40 mV in 20 mV increments) were applied for 500 ms from a holding voltage of −10 mV, using a Labmaster TL40 interface and pClamp 5.6 software (Axon Instruments Inc., Union City, CA, USA). Whole-cell currents were measured twice, filtered at 100 Hz, averaged, and stored for later analysis. The protocol was repeated after exposing oocytes to 10 μmol/l amiloride for 30 s. This relatively high concentration of amiloride was used to ensure maximal inhibition of whole-cell Na^+^ currents in oocytes expressing hENaC (Canessa et al. 1993). Differences between the pre- and post-amiloride whole-cell currents at each command voltage were taken to reflect whole-cell Na^+^ currents. After washing off amiloride, an additional set of whole-cell current measurements were obtained at each command voltage.

### Agents Used to Study hENaC Regulation by PKC

To determine the effects of PKC, amiloride-sensitive whole-cell currents were measured using the above protocol, first in the absence and then after exposing the oocytes to 500 nmol/l PMA for 30 min to activate PKC. In preliminary experiments, using oocytes expressing wild-type hENaC, the changes in amiloride-sensitive whole-cell currents produced by PMA had reached a steady-state at this time point. To provide ‘negative control’ data, similar experiments were performed with 500 nmol/l phorbol didecanoate (PDD), an inactive phorbol ester which does not stimulate PKC. Additional experiments were done with a combination of 500 nmol/l PMA and 50 µmol/l calphostin C, a specific inhibitor of PKC (Hartzell and Rinderknecht 1996). ‘Time control’ experiments were performed with cDNA-injected oocytes to exclude possible ‘run-down’ of the whole-cell Na^+^ currents.

### Statistical Analyses

Data are shown as mean ± SEM. Normalized amiloride-sensitive whole-cell Na^+^ currents at −100 mV were compared using either Student’s *t* test (for two sets of data), or one-way ANOVA with post hoc analysis (for more than two sets of data). Current–voltage relationships were compared using repeated measures ANOVA. *P* < 0.05 was taken to indicate a statistically significant difference between two mean values. Data were analyzed using SPSS for Windows (release 10.1).

## Results

### Effect of PKC on Wild-Type Human Renal ENaC

**Fig. 1:**
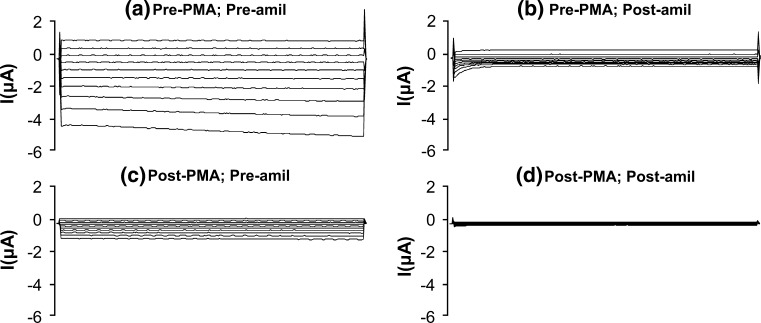
Whole-cell currents (command voltages applied for 500 ms between −140 and +40 mV in 20 mV increments) in an oocyte expressing wild-type hENaC **a** in the absence of PMA and amiloride, **b** in the absence of PMA but in the presence of amiloride, **c** in the presence of PMA but in the absence of amiloride, and **d** in the presence of both PMA and amiloride. PMA markedly decreased oocyte amiloride sensitivity, consistent with inhibition of hENaC

Initial experiments were done in oocytes expressing wild-type hENaC to evaluate the changes in amiloride-sensitive whole-cell currents produced by 30 min exposure to 500 nmol/l PMA. Amiloride largely abolished the pre-PMA whole-cell currents (Fig. 1a, b), consistent with Na^+^ channel inhibition. In the absence of amiloride, post-PMA whole-cell currents were markedly reduced compared with the pre-PMA whole-cell currents (Fig. 1a, c), and the residual currents were abolished by amiloride (Fig. 1c, d). With each oocyte, normalization of the post-PMA data to data obtained in the basal (pre-PMA) state indicated that PMA decreased amiloride-sensitive Na^+^ currents in oocytes expressing wild-type hENaC by 80 ± 3 % at −100 mV (*n* = 23; *P* < 0.001), whereas there was no significant change in cDNA-injected ‘time control’ oocytes (*n* = 12). These results were in agreement with those of previous studies, which demonstrated that PKC activation significantly decreased amiloride-sensitive Na^+^ channel activity (Ling et al. 1992; Awayda et al. 1996; Awayda 2000).

### Effect of PDD on Wild-Type Human Renal ENaC

**Fig. 2:**
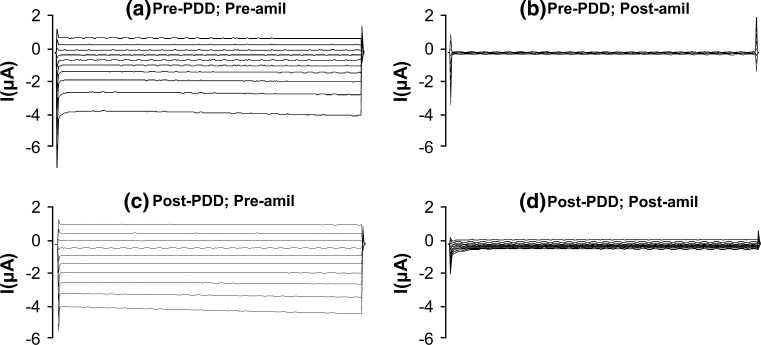
Whole-cell currents (command voltages applied for 500 ms between −140 and +40 mV in 20 mV increments) in an oocyte expressing wild-type hENaC **a** in the absence of PDD and amiloride, **b** in the absence of PDD but in the presence of amiloride, **c** in the presence of PDD but in the absence of amiloride, and **d** in the presence of both PDD and amiloride. PDD had no effect on oocyte amiloride sensitivity

‘Negative control’ experiments were done in oocytes expressing wild-type hENaC to evaluate amiloride-sensitive Na^+^ currents after 30 min exposure to 500 nmol/l PDD, an inactive phorbol ester which does not activate PKC. As expected, amiloride markedly decreased the pre-PDD whole-cell currents (Fig. 2a, b). In the absence of amiloride, post-PDD whole-cell currents were similar to the pre-PDD whole-cell currents (Fig. 2a, c), but decreased markedly with the subsequent addition of amiloride (Fig. 2c, d). Normalization of the post-PDD data to data obtained in the basal (pre-PDD) state indicated that PDD had no significant effect on amiloride-sensitive Na^+^ currents in oocytes expressing wild-type hENaC (*n* = 6).

### Effect of PMA + Calphostin C on Wild-Type Human Renal ENaC

**Fig. 3:**
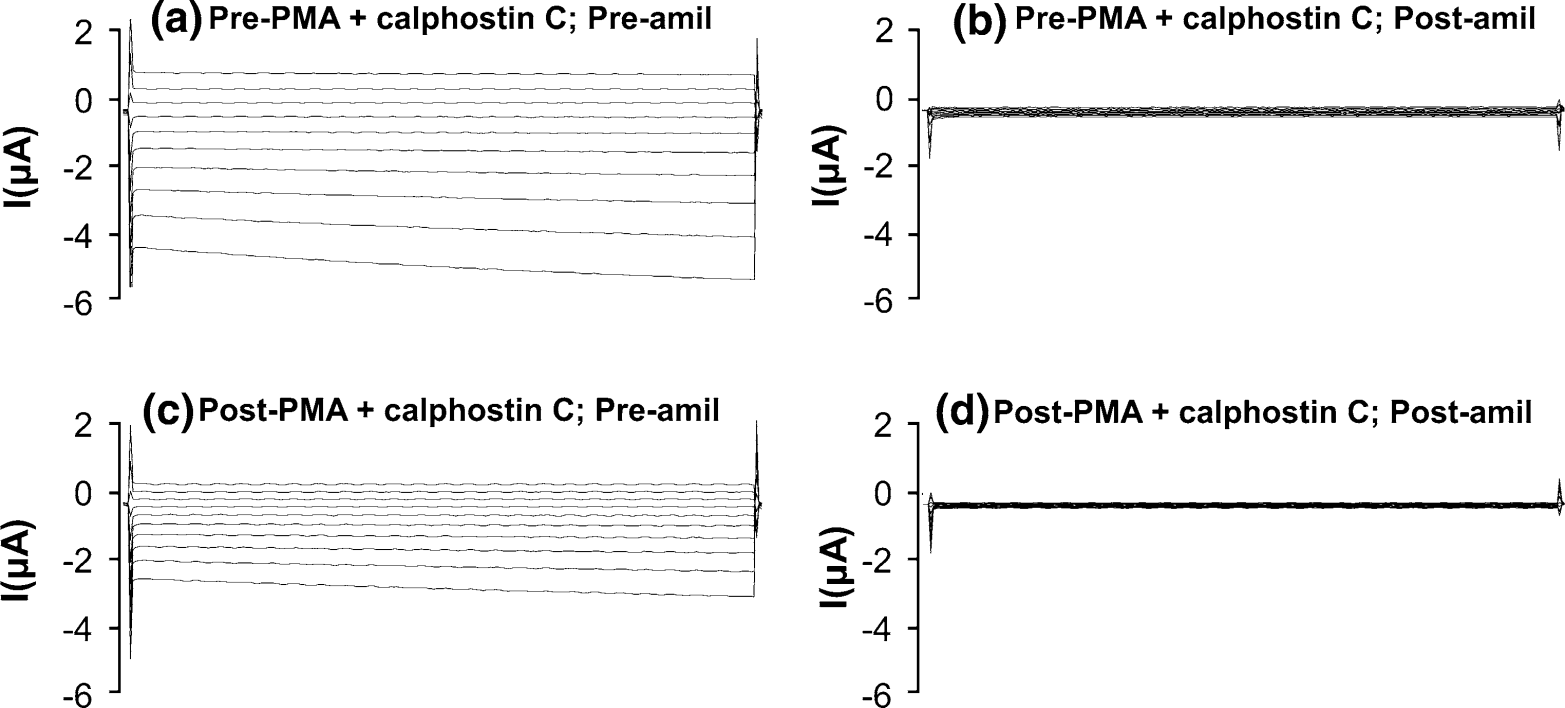
Whole-cell currents (command voltages applied for 500 ms between −140 and +40 mV in 20 mV increments) in an oocyte expressing wild-type hENaC **a** in the absence of PMA + calphostin C and amiloride, **b** in the absence of PMA + calphostin C but in the presence of amiloride, **c** in the presence of PMA + calphostin C but in the absence of amiloride, and **d** in the presence of both PMA + calphostin C and amiloride. Calphostin C significantly reduced the decrease in oocyte amiloride sensitivity produced by PMA alone (see Fig. 1 for comparison)

In further experiments, the protocol with PMA in oocytes expressing wild-type hENaC was repeated with both 500 nmol/l PMA and 50 μmol/l calphostin C (a specific PKC inhibitor) in the superfusing solution. Whereas PMA alone markedly decreased whole-cell currents in the absence of amiloride (Fig. 1a, c), this effect was attenuated when PMA was applied in the presence of calphostin C (Fig. 3a, c). The combination of PMA and calphostin C produced a 35 ± 7 % decrease in amiloride-sensitive Na^+^ current at −100 mV (*n* = 8, *P* < 0.03), and one-way ANOVA and post hoc analyses indicated that this change was significantly less than the 80 ± 3 % decrease produced by PMA alone (*P* < 0.001). The decrease in amiloride-sensitive Na^+^ current produced by PMA and calphostin C also differed significantly (*P* < 0.001) from the small decrease in Na^+^ current in the ‘time control’ experiments (Fig. 5a). Thus, our results indicate that calphostin C had a substantial inhibitory effect on the ability of PMA to decrease wild-type Na^+^ channel activity.

### Effect of PMA on Liddle-Mutated Human Renal ENaC

**Fig. 4:**
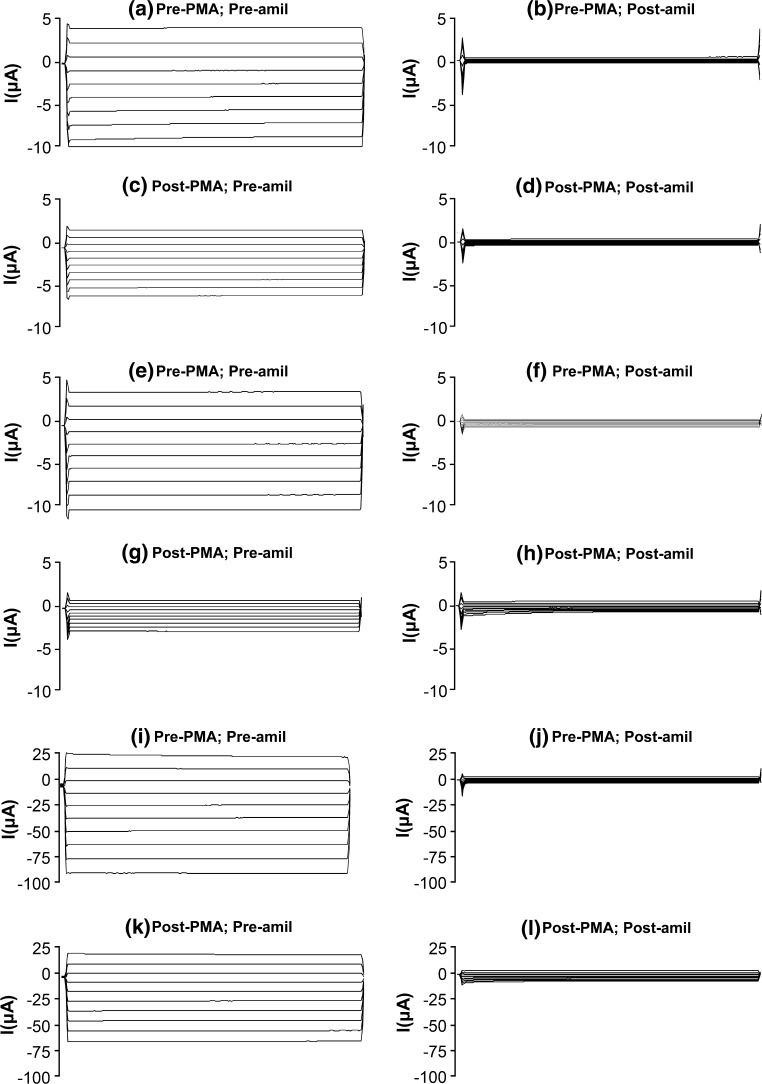
Whole-cell currents (command voltages applied for 500 ms between −140 and +40 mV in 20 mV increments) in oocytes expressing **a**–**d** β-subunit Liddle-mutated hENaC, **e–h** γ-subunit Liddle-mutated hENaC, or **i**–**l** both β- and γ subunit Liddle-mutated hENaC, **a**, **e**, **i** in the absence of PMA and amiloride, **b**, **f**, **j** in the absence of PMA but in the presence of amiloride, **c**, **g**, **k** in the presence of PMA but in the absence of amiloride, and **(d, h, l)** in the presence of both PMA and amiloride

Since the COOH termini of wild-type hENaC β- and γ-subunits contain potential PKC phosphorylation sites (Shimkets et al. 1998), additional experiments were done to evaluate the effect of PMA on hENaC containing the Liddle-mutated β- and γ-subunits. The small and insignificant decreases in amiloride-sensitive Na^+^ currents seen with wild-type hENaC in ‘time control’ oocytes and those exposed to PDD were identical, and thus only ‘time controls’ were used for the experiments with Liddle-mutated hENaC. Basal amiloride-sensitive Na^+^ currents in oocytes expressing the Liddle-mutated β-subunit (Fig. 4a, b; *n* = 14) or the Liddle-mutated γ-subunit (Fig. 4e, f; *n* = 16) were substantially greater than those expressing wild-type hENaC (Fig. 1a, b), as reported previously (Hansson et al. 1995; Robins et al. 2013; Robins and Sandle 2014). Furthermore, basal amiloride-sensitive Na^+^ currents in oocytes expressing both the Liddle-mutated β- and the γ-subunits (Fig. 4i, j; *n* = 9) were almost an order of magnitude greater than in those expressing the Liddle-mutated β- or the γ-subunit alone, as reported by others (Hansson et al. 1995).

In the absence of amiloride, PMA decreased whole-cell currents in oocytes expressing the Liddle-mutated β-subunit (Fig. 4a, c), but to a smaller degree than in oocytes expressing wild-type hENaC (Fig. 1a, c), Nevertheless, at −100 mV, PMA elicited a significant 54 ± 4 % (*n* = 14; *P* < 0.001) decrease in amiloride-sensitive whole-cell Na^+^ currents in oocytes expressing the Liddle-mutated β-subunit (Fig. 4a–d). One-way ANOVA followed by post hoc analyses indicated that the decrease in response to PMA in oocytes expressing the Liddle-mutated β-subunit (54 ± 4 %) was significantly less than that in oocytes expressing wild-type hENaC (80 ± 3 %; *P* < 0.0025). In the absence of amiloride, PMA also decreased whole-cell currents in oocytes expressing the Liddle-mutated γ-subunit (Fig. 4e, g), almost to the same degree as that seen in oocytes expressing wild-type hENaC (Fig. 1a, c). At −100 mV, PMA elicited a 68 ± 5 % decrease (*n* = 16; *P* < 0.001) in amiloride-sensitive whole-cell Na^+^ currents in oocytes expressing the Liddle-mutated γ-subunit (Fig. 4e–h), and one-way ANOVA followed by post hoc analyses indicated that this decrease was similar to that in oocytes expressing wild-type hENaC (80 ± 3 %; *P* = 0.10).

Additional experiments were performed using oocytes co-expressing both the Liddle-mutated β-subunit and the Liddle-mutated γ-subunit of hENaC. In the absence of amiloride, PMA decreased whole-cell currents in these oocytes (Fig. 4i, k) almost to the same degree as that seen in oocytes expressing the Liddle-mutated β-subunit alone (Fig. 4a, c). At −100 mV, PMA elicited a significant 37 ± 5 % (*n* = 9; *P* < 0.0025) decrease in amiloride-sensitive whole-cell Na^+^ currents in oocytes co-expressing the Liddle-mutated β- and γ-subunits (Fig. 4i–l). One-way ANOVA followed by post hoc analyses indicated that the PMA-induced decrease in amiloride-sensitive whole-cell Na^+^ currents in oocytes co-expressing the Liddle-mutated β- and γ-subunits was significantly less than in oocytes expressing wild-type hENaC or the Liddle-mutated γ-subunit alone (*P* < 0.002 in both cases), but was similar to the decrease in oocytes expressing the Liddle-mutated β-subunit alone (*P* = 0.08).

### Summary of Wild-Type Human Renal ENaC Data

**Fig. 5:**
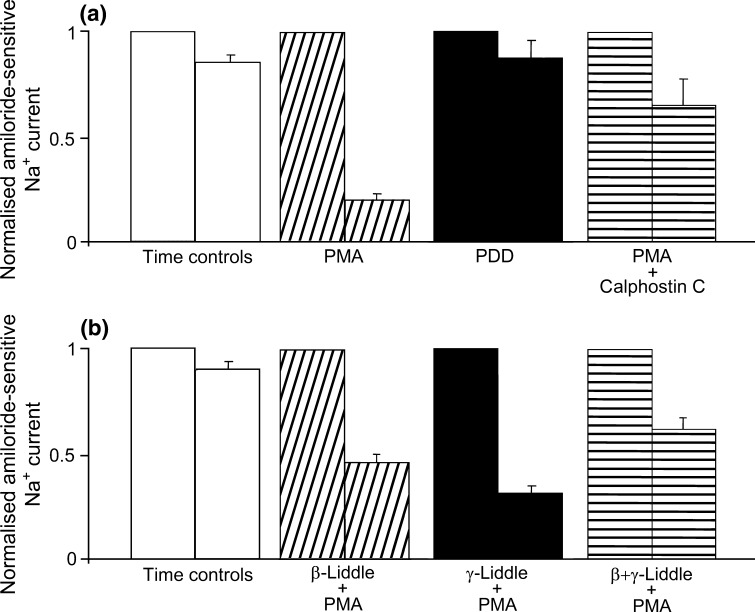
Summary of amiloride-sensitive whole-cell Na^+^ currents (recorded at −100 mV) in oocytes **a** expressing wild-type hENaC in time controls (*open bars, n* = *12*), and 30 min after the addition of PMA (*diagonally hatched bars, n* = *23*), PDD (*solid bars, n* = *6*), or PMA+ calphostin C (*horizontally hatched bars, n* = *8*), and **b** expressing Liddle-mutated β-subunit (*diagonally hatched bars, n* = *14*), Liddle-mutated γ-subunit (*solid bars, n* = *16*), or both Liddle-mutated β-and γ-subunits (*horizontally hatched bars, n* = *9*), 30 min after the addition of PMA, with accompanying time controls (*open bars, n* = *4*)

Normalized amiloride-sensitive Na^+^ currents from each protocol in oocytes expressing wild-type hENaC are summarized in Fig. 5a. In each case, the left-hand bar indicates normalized data at time zero in ‘time control’ experiments, and in the presence of PMA alone, PDD, or PMA + calphostin C. In contrast to the ‘time control’ experiments, PMA elicited a 80 ± 3 % decrease in amiloride-sensitive Na^+^ current (*P* < 0.001), whereas there was no significant change with PDD (*P* = 0.121), and PMA + calphostin C elicited a smaller but significant 35 ± 7 % decrease in amiloride-sensitive Na^+^ current (*P* < 0.03) compared with PMA alone. One-way ANOVA and post hoc analyses indicated that, compared with the response to PMA alone, those with PMA + calphostin and PDD were significantly different (*P* < 0.001 in both cases). Taken together, these results indicate that the inhibitory effect of PMA on wild-type hENaC is mediated by activation of PKC rather than via a nonspecific effect of the phorbol ester.

### Summary of Liddle-Mutated Human Renal ENaC Data

Normalized amiloride-sensitive Na^+^ currents from oocytes expressing the Liddle-mutated β-subunit, the Liddle-mutated γ-subunit, or both the Liddle-mutated β-subunit and the Liddle-mutated γ-subunit, are summarized in Fig. 5b. In each case, the left-hand bar indicates normalized data at time zero in the three groups of oocytes expressing the Liddle-mutated subunits treated with PMA. In contrast to the ‘time control’ experiments, PMA elicited decreases in amiloride-sensitive Na^+^ current in oocytes expressing the Liddle-mutated β-subunit, the Liddle-mutated γ-subunit, or both the Liddle-mutated β-subunit and the Liddle-mutated γ-subunit of 54 ± 4 % (*P* < 0.001), 68 ± 5 % (*P* < 0.001), and 37 ± 5 % (*P* < 0.0025), respectively. One-way ANOVA followed by post hoc analyses indicated that, compared with the PMA-induced decrease in amiloride-sensitive Na^+^ current in oocytes expressing wild-type hENaC (80 ± 3 %), the decreases in oocytes expressing Liddle-mutated β-subunit (54 ± 4 %), or both the Liddle-mutated β-subunit and the Liddle-mutated γ-subunit (37 ± 5 %) were significantly less (*P* < 0.0025 and *P* < 0.002, respectively). By contrast, the decrease in oocytes expressing the Liddle-mutated γ-subunit (68 ± 5 %) was similar to that in oocytes expressing wild-type hENaC (*P* = 0.1).

## Discussion

Previous studies in Madin-Derby canine kidney (MDCK) cells stably transfected with rat ENaC α-, β-, and γ-subunits have shown that whereas the β- and γ-subunits (but not the α-subunits) are phosphorylated under basal conditions, PKC enhances phosphorylation of the β- and γ-subunits but not the α-subunit, the sites of phosphorylation being serine and threonine residues within the COOH termini (Shimkets et al. 1998). Putative PKC phosphorylation sites are located close to the PY motifs in the COOH termini of ENaC β- and γ-subunits (Barbry and Hofman 1997), raising the possibility that ENaC containing Liddle-mutated β- and/or γ-subunits mutations might have an altered response to PKC activation. In the present study, we explored this possibility by studying the effect of PMA-activated PKC on amiloride-sensitive Na^+^ currents in oocytes expressing wild-type hENaC, and hENaC containing the Liddle-mutated β- and γ-subunits, either alone or in combination. We found that PMA reduced amiloride-sensitive Na^+^ currents by 80 % in oocytes expressing wild-type hENaC, whereas the inactive phorbol ester PDD had no effect. In addition, the inhibitory effect of PMA-activated PKC on amiloride-sensitive Na^+^ currents was attenuated by the PKC inhibitor calphostin C, the 35 % decrease being significantly less (*P* < 0.001) than that produced by PMA alone. Thus, from these results, it is clear that PMA markedly decreased wild-type hENaC though a mechanism involving the activation of PKC. Our findings are consistent with those reported in renal A6 cells, in which PKC down-regulated ENaC by phosphorylating the β- and γ-subunits, but not the α-subunit (Stockand et al. 2000). PKC decreases both open channel probability (Ling and Eaton 1989; Ling et al. 1992; Oh et al. 1993; Awayda et al. 1996) and channel density (Els et al. 1998) of wild-type ENaC, and similar changes may underlie the PMA-induced 80 % decrease in amiloride-sensitive Na^+^ current we observed in oocytes expressing wild-type hENaC, following PKC-mediated phosphorylation of the β- and/or γ-subunits. We have previously shown that raising intracellular Ca^2+^ in oocytes expressing wild-type hENaC also decreases amiloride-sensitive Na^+^ current by 55 % through a W-7-sensitive (i.e., calmodulin-dependent) mechanism (Robins and Sandle 2014). However, it seems unlikely that the inhibitory effect of PMA-activated PKC on the amiloride-sensitive Na^+^ currents was Ca^2+^ dependent, since PKC has Ca^2+^-independent effects in other cell types (Iacopetta et al. 1986; Bonaccorsi et al. 1998).

By contrast, in oocytes expressing the Liddle-mutated β-subunit, PMA decreased amiloride-sensitive Na^+^ currents by only 54 %, a change significantly less than that seen in those expressing wild-type hENaC (80 %; *P* < 0.0025). Furthermore, in oocytes expressing the Liddle-mutated γ-subunit, PMA decreased amiloride-sensitive Na^+^ currents by 68 %, a change that was not significantly different from the 80 % decrease seen in oocytes expressing wild-type hENaC (*P* = 0.1). In oocytes expressing hENaC containing both the Liddle-mutated β- and the Liddle-mutated γ-subunits, PMA significantly decreased amiloride-sensitive Na^+^ currents by 37 % (*P* < 0.0025), a change similar (*P* = 0.08) to that seen in oocytes expressing hENaC containing the Liddle-mutated β-subunit alone. Previous studies have indicated that PKC down-regulates both the β- and γ-subunits of wild-type hENaC (Stockland et al. 2000), presumably at the putative PKC phosphorylation sites present close to the PY motifs of their COOH termini (Barbry and Hofman 1997). Contrary to the suggestion that the β-subunit consensus site may be a poorer substrate for PKC-induced phosphorylation than the corresponding site on the γ-subunit (Barbry and Hofman 1997), our results indicate that it is the inclusion of the Liddle-mutated β-subunit, rather than the Liddle-mutated γ-subunit, which diminishes PKC’s inhibitory effect on hENaC.

An additional intracellular signaling mechanism to be considered when interpreting our data is that involving myristolated alanine-rich C kinase substrate (MARCKS) (Sengupta et al. 2007). MARCKS possesses sites for PKC phosphorylation (Hartwig et al. 1992) and acts as an adaptor protein, binding to and presenting phosphatidylinositol phosphates (PIPs; particularly phosphatidylinositol 4,5-biphosphate, PIP2) to regulate ENaC activity (Alli et al. 2012). In *Xenopus* 2F3 renal epithelial cells, MARCKS colocalizes with PIP2 at the apical membrane, but translocates to the cytoplasm after PKC stimulation, so that PKC-induced MARCKS phosphorylation decreases amiloride-sensitive Na^+^ currents; conversely, inhibition of PKC-promoted binding of unphosphorylated MARCKS to PIPs for presentation to ENaC increases amiloride-sensitive Na^+^ currents (Alli et al. 2012). Thus, in our experiments with hENaC (particularly the wild-type), PMA-activated PKC could have promoted translocation into the cytoplasm of MARCKS present in the oocyte membrane, thereby decreasing PIP-regulated hENaC activity.

Thus, we have shown that PKC produces substantial inhibition (80 %) of amiloride-sensitive Na^+^ currents through wild-type hENaC, and Na^+^ currents through hENaC containing the Liddle-mutated γ-subunit are inhibited to a similar extent (68 %). By contrast, PKC produces much less inhibition (54 %) of Na^+^ currents through hENaC containing the Liddle-mutated β-subunit, which suggests that the COOH-terminus of this subunit (rather than the γ-subunit) possesses one or more phosphorylation sites, which are critical for the regulation of wild-type hENaC by PKC. Whether or not this apparent difference between the susceptibilities of the Liddle-mutated β-subunit and the Liddle-mutated γ-subunit to PKC has any clinical relevance is unclear, because the identification of specific Liddle β- and γ-mutations has generally been restricted to individual patients or clusters of patients in a single family. However, a recent genetic study of 330 young Chinese hypertensive patients identified Liddle syndrome in five (1.52 %), and in 12 of their relatives (Wang et al. 2015). Of the five patients, four had Liddle β-subunit mutations, whereas only one had a Liddle γ-subunit mutation. If the β-subunit mutation predominates to a similar degree in cases of Liddle syndrome irrespective of ethnicity, it is possible that a significant impairment of PKC-induced down-regulation of ENaC contributes to the salt-sensitive hypertension that characteristically occurs in these patients.
